# Unexplained neoplastic anastomotic recurrence after right hemicolectomy: a case report

**DOI:** 10.1186/s13256-020-02529-z

**Published:** 2020-10-20

**Authors:** Pietro Genova, Vincenzo Davide Palumbo, Attilio Ignazio Lo Monte, Calogero Cipolla, Gaspare Genova

**Affiliations:** 1 Department of Surgical, Oncological and Oral Sciences, University of Palermo, Via del Vespro 129, 90127 Palermo, Italy; 2 Euro-Mediterranean Institute of Science and Technology, Palermo, Italy

**Keywords:** Anastomotic recurrence, Case report, Colon carcinoma, Right hemicolectomy

## Abstract

**Background:**

Anastomotic recurrences of the colon are postulated to arise due to inadequate margins, tumor implantation by exfoliated cells, altered biological properties of bowel anastomosis, and missed synchronous lesions. In this paper, a case of unexpected early local recurrence after surgery for colon cancer is presented.

**Case presentation:**

A 68-year-old Caucasian man underwent right hemicolectomy for invasive G2 adenocarcinoma. Two months later, endoscopy revealed a wide and well-functioning anastomosis with a hyperemic, hard, and thickened mucosal area of about 2 cm in diameter. Biopsies showed the presence of an adenocarcinoma with the same grading of the previous lesion. Ten days later, the patient underwent a new intervention; the last 10 cm of the ileum and half of the remaining transverse colon were resected, and the patient started adjuvant therapy. Specimen examination confirmed the presence of an adenocarcinoma (G2) penetrating the muscular layer of the wall; also, in this case, resection edges were free from tumoral invasion, and the removed lymph nodes were exempt from neoplastic colonization. The patient was seen in follow-up for about 5 years, and he did not show local or systemic manifestations.

**Conclusions:**

Whenever a neoplastic recurrence on the anastomotic line occurs, in the presence of negative intestinal margins, as usual in right colectomies, the implantation of neoplastic cells could be the possible cause.

## Introduction

Anastomotic recurrence (AR) after resection for colorectal cancer can assume a wide range of presentations. In the past, AR represented a clinical challenge: Data from 1948 to 1976 reported rates between 10% and 35% [1, 2]; more recently, this range has narrowed, with significantly lower rates being due to available diagnostic and therapeutic improvements [3]. Tumor recurrence is also more frequent after surgery in the left colon and rectum than in surgery in the ascending colon. Rates reported in the literature range from 7.8% to 13% in the former [4, 5] and from 0.8% to 14% [6, 7] in the latter. The rate of success in AR is relatively low, likely due to delayed diagnoses [2]. The mortality rate is 10–15% [8–11]. AR is thought to be caused by inadequate resection margins or by implantation of exfoliated cancer cells. Viable tumor cells, shed from the surface of solid tumor tissue in the lumen of the colon or rectum during surgery, may be responsible. Alternative mechanisms include metachronous carcinogenesis at perianastomotic sites with proliferative instability, and adaptive hyperplasia of the epithelium at the suture line might play an important role. In this report, we describe an unexpected case of AR soon after curative right colectomy with ileocolic anastomosis performed to treat adenocarcinoma of the cecum.

## Case presentation

**Fig. 1:**
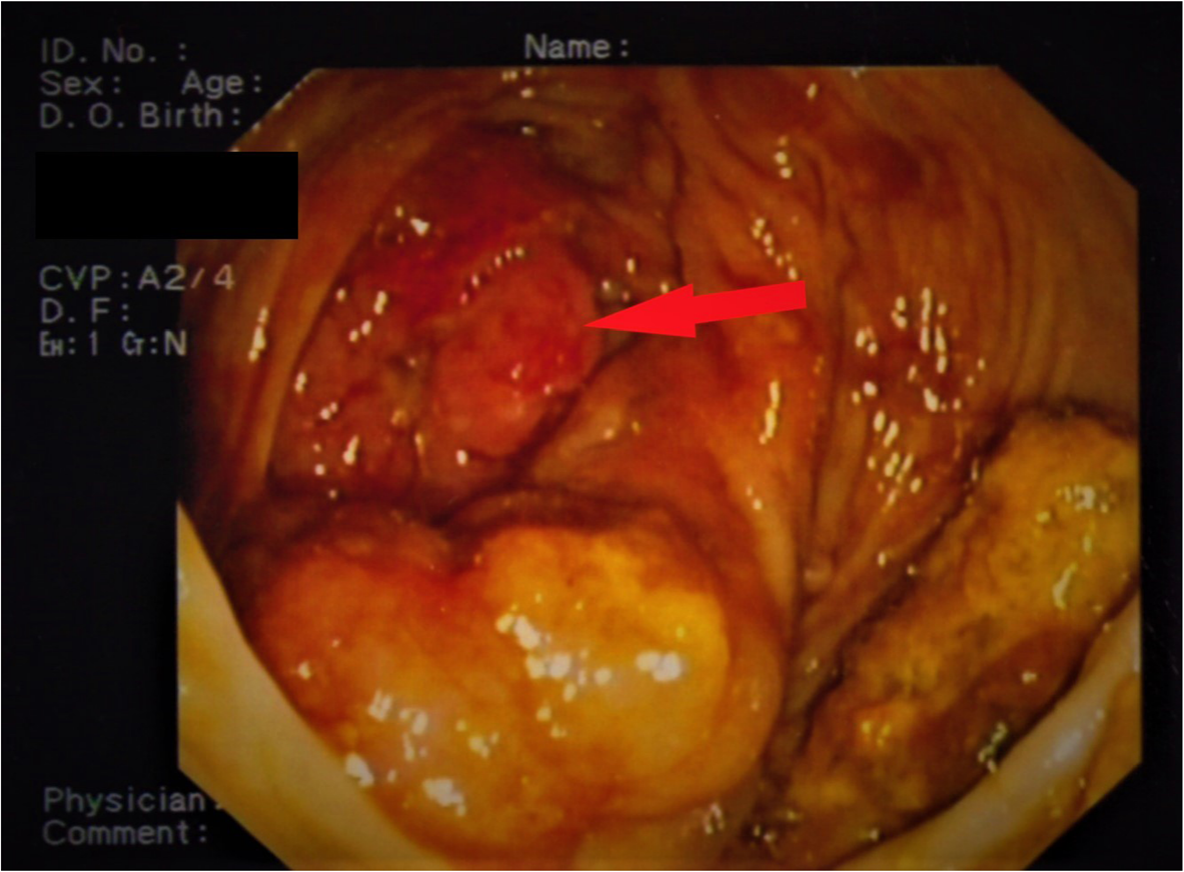
Endoscopic image of the cecum. The *red arrow* indicates a tan, 4-cm-wide neoformation with small foci of hemorrhage

**Fig. 2:**
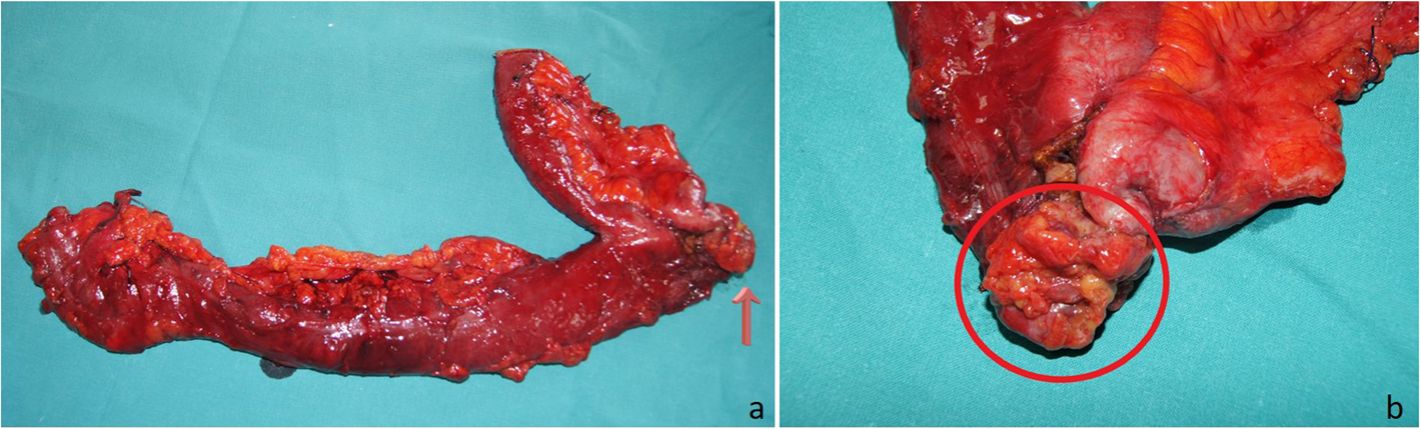
Right hemicolectomy postoperative specimen. The *red arrow* in (**a**) and the *red circle* in (**b**) indicate the neoplastic mass

A 68-year-old Caucasian man with no relevant medical or surgical history or colorectal cancer family history came to our department with microcytic anemia of unknown origin. In order to understand the primary cause of the presented condition, he underwent several clinical investigations. Endoscopy allowed us to detect a tan, 4-cm-wide neoformation of the cecum with small foci of hemorrhage (Fig. 1). Biopsies were received from the mucosal lesion. A few days later, a histological diagnosis of invasive G2 adenocarcinoma was made. The patient was admitted to our unit for surgical treatment. He underwent right hemicolectomy (Fig. 2) with ileocolic, side-by-side isoperistaltic anastomosis after intestinal preparation with 4 L of polyethylene glycol (PEG) the night before surgery. Soon after intestine removal, some gauzes soaked in polyvinylpyrrolidone 5% were inserted into the open abdomen for 15 minutes in order to drape the bowel stumps and avoid fecal leakage. The anastomosis was hand-sewn with extramucosal, separated, 2-0 absorbable stitches. The patient’s postoperative course was normal, and he was discharged 6 days after surgery.

**Fig. 3:**
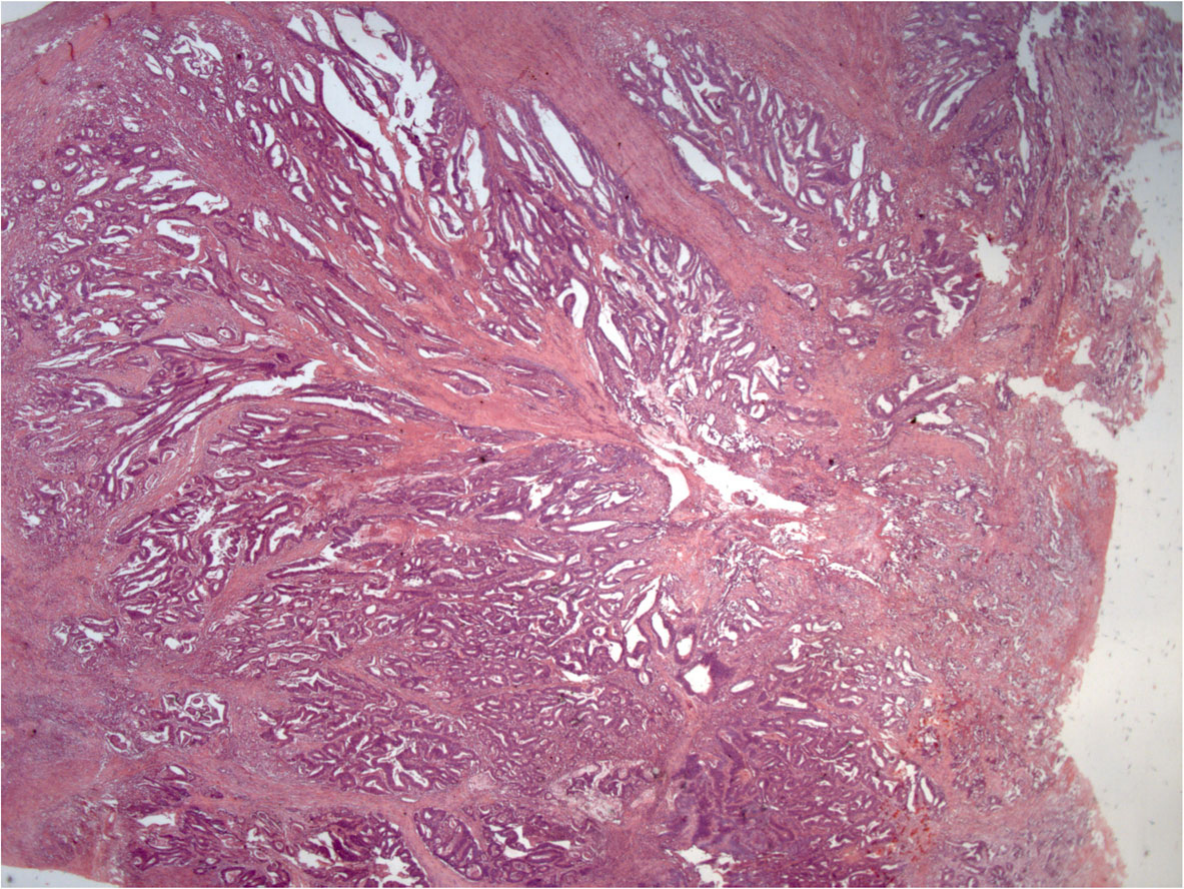
Neoplastic polypoid lesion of the cecum. Histology revealed a moderately differentiated adenocarcinoma. The tumor invades the muscularis propria and subserosa (Hematoxylin and eosin; original magnification, 12 ×)

**Fig. 4:**
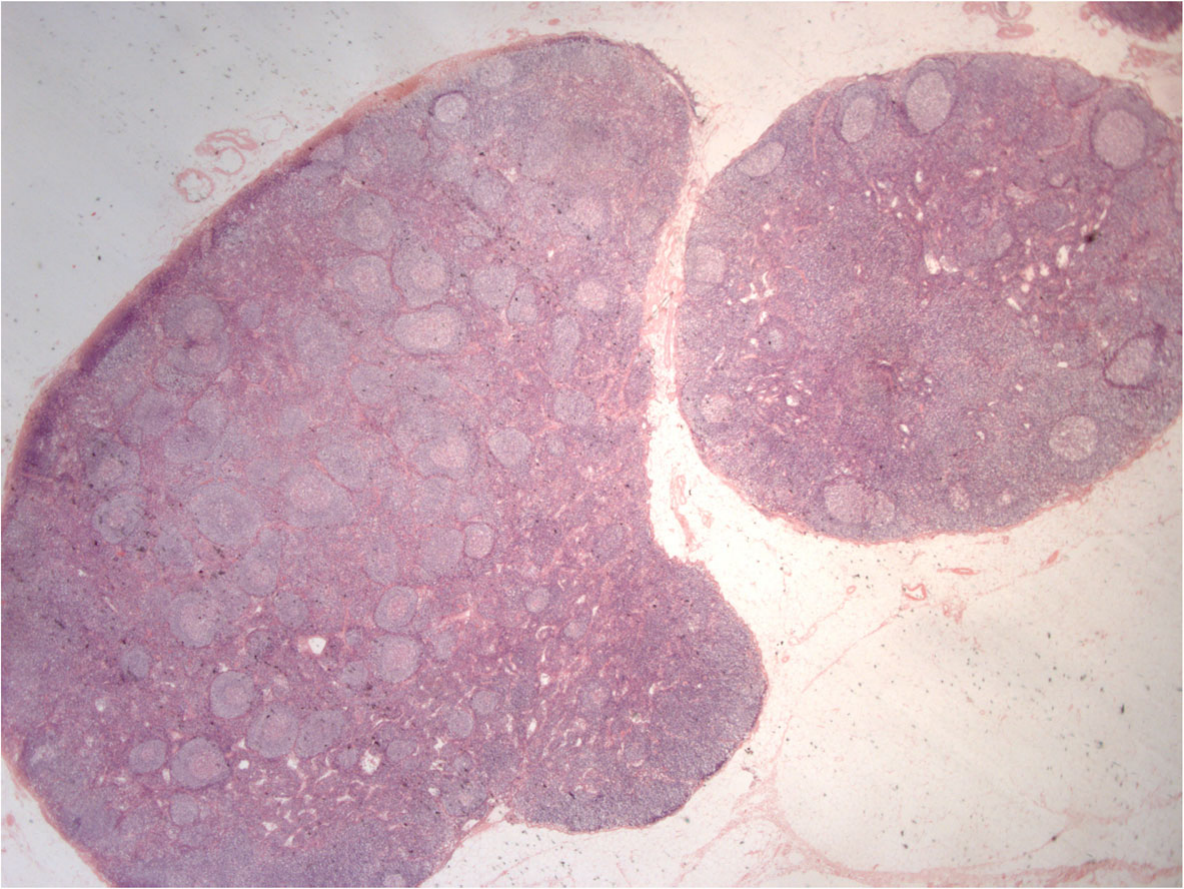
Neoplastic polypoid lesion of the cecum. Reactive lymph nodes are shown (Hematoxylin and eosin; original magnification, 12 ×)

Histology of the specimen showed the presence of adenocarcinoma (G2) invading the whole intestinal wall and extending to the fibroadipose perivisceral layers. The resection edges were unharmed, and the lymph nodes were exempt from metastatic colonization (Figs. 3, 4). The patient refused adjuvant chemotherapy, and he was referred to a program for endoscopic follow-up.

**Fig. 5:**
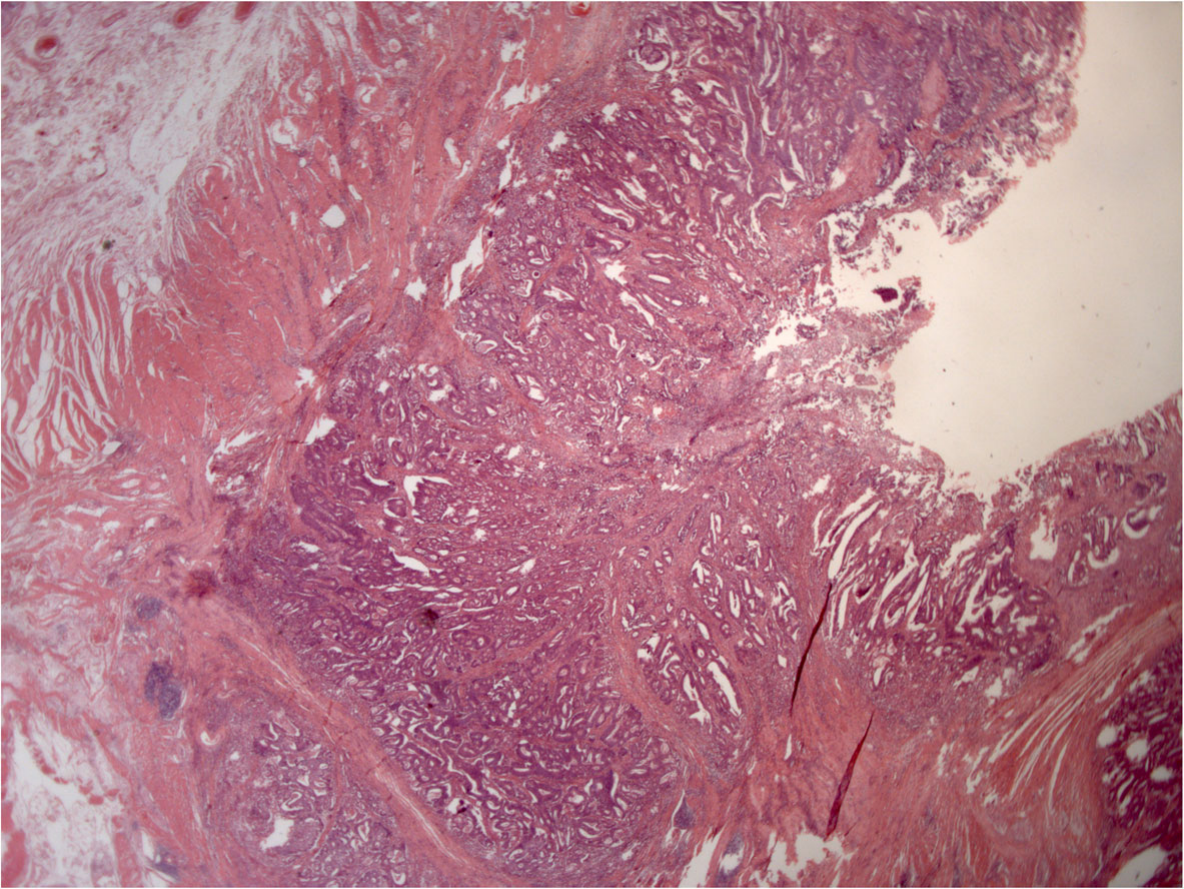
Anastomotic recurrence 2 months after surgery. Moderately differentiated adenocarcinoma is shown. Muscularis propria invasion can be seen (Hematoxylin and eosin; original magnification, 12 ×)

Two months later, endoscopy revealed a wide and well-functioning anastomosis with a hyperemic, hard, and thickened mucosal area of about 2 cm in diameter. Biopsies showed the presence of an adenocarcinoma with the same grading of the previous lesion. Ten days later, the patient underwent a new operation; the last 10 cm of the ileum and half of the remaining transverse colon were resected, and the patient started adjuvant therapy. Specimen examination confirmed the presence of adenocarcinoma (G2) penetrating the muscular layer of the wall; also, in this case, the resection edges were free from tumoral invasion, and the removed lymph nodes were exempt from neoplastic colonization (Fig. 5). The patient was seen in follow-up for about 5 years, and he did not show local or systemic manifestations.

## Discussion and conclusions

ARs can be the expression of a systemic disease or a local manifestation. Most of the local recurrences that occur after 2 years should be considered as metastatic recurrences (peritoneum or soft tissue) or as second primary lesions. Local recurrence occurring before 2 years is due to inadequate excision with positive margins, penetration of the lymphatic vessels, implantation of viable neoplastic cells, or changes in the biology of the tumor or of the colonic mucosa. Even a missed synchronous lesion can present as a recurrent tumor at a later stage.

Another possible cause of local recurrence could be the presence of a small synchronous neoplasia, unknown at the moment of resection but close to the anastomosis, as in case of a small, flat lesion. Synchronous colorectal cancer (SCCR) diagnosis can be made when it occurs simultaneously or within 6 months from the initial diagnosis and the histopathological criteria of Warren and Gates are respected: Each tumor has to show a clear outline of malignancy, has to be clearly distinct from the other, and must not be the metastatic expression of the other.

SCCR prevalence ranges from 2.3% to 12.4% [12, 13]. Because these data refer to old records, such a wide range is likely amenable to the different diagnostic and therapeutic means but also to the scarce number of cases reported and to a not always available follow-up. SCCR is more common in elderly patients and affects the right colon in most of these cases [13–17]. Certainly, a large body of data has confirmed a higher incidence of synchronous adenomatous lesions in patients with SCCR than in patients with a single lesion [13–22]. In our patient’s case, SCCR could be excluded because even a small, flat mucosal lesion would have been detected during follow-up, and, overall, it would not have been escaped to the histology of the resected intestinal stumps.

Whenever a lesion appears over a period of 24 months after surgery, it can be classified as a second primary neoplasia (metachronous). A relevant number of studies, however, report an interval of 32–38 months before a malignant lesion occurs [23–25].

Data extracted from the California Cancer Registry comprising a large population have shown that a metachronous cancer develops in about 32 months and, in 50% of the cases, in more than 2.5 years. In some cases, malignancies have been discovered 3 years after surgery [24]. More recent data suggest a lower rate of detection (about 15%) [26]. The onset of a metachronous cancer on an anastomosis line could be connected both to the development of a new neoplastic process and to an alteration of biological properties of the mucosa. Likely, especially in those cases of healing impairment, the continuous proliferative stimulus due to chronic inflammation could favor neoplastic growth. Interestingly, the risk of developing a second primary tumor seems to increase in patients undergoing surgery for a neoplasia of the transverse or descending colon [24, 26, 27]. A second primary neoplasia could also be explained by an alteration of mucosal biological properties. In particular, in right hemicolectomies, the absence of the ileocolic valve can influence the biology of the anastomotic mucosa, as indirectly proved by the existence of the so-called diversion colitis. Considering the relatively early occurrence of the recurrence, a metachronous lesion can be categorically excluded.

Most local recurrences can be explained by considering insufficient resection edges or hidden involvement of the lymphatics, but they could also be due to the implantation of exfoliated neoplastic cells into the intestinal wall, favored by surgical manipulations.

A large number of local recurrences are more frequent after anterior resection of the rectum and more often develop from pararectal tissues, involving subsequently the anastomotic line, and are more frequent when an anastomotic dehiscence or local infection occurs. In a large number of cases, AR can be attributed to an insufficient size of the resection due to the difficulty in extending as much as possible the intestinal section beyond the neoplastic lesion. In the right colon, the possibility of greater extension of the resection edges behind and beyond the neoplastic lesion allows ruling out an insufficient extension of the resection as a cause.

Implantation on the anastomotic line of exfoliated viable neoplastic cells could be considered as an additional factor in AR development. The possibility that exfoliated viable cells grow on the colic mucosa far from the primitive lesion has been fully proved also by experimental studies. Tumoral cells are often recognized in the abdominal washing liquid. This possibility, which could be favored by surgical manipulation, has been proved also by experiments involving animals. In addition, neoplastic cell homing could be favored by damaged mucosa, as in case of anastomotic margins. Importantly, neoplastic cell seeding does not depend on tumoral dimensions or neoplastic invasiveness. In order to prevent possible implantation of exfoliated cells, intestinal irrigation with cancericidal substances has been examined. This technique seems to have contributed to a significant reduction in recurrence rates in cases of anterior resection.

The application of a water-based solution of polyvinylpyrrolidone for 5 minutes is usually preferred, although longer periods (15 minutes) seem to give better results; periods longer than 30 minutes damage the cells of the normal colic mucosa irreversibly.

These methods seem to reduce the incidence of recurrence on the anastomotic line from 10–16% to 2–3%. At the beginning of 1947, in order to reduce the risk of relapse, Lloyd Davis and Naunton Morgan already decided to irrigate the colon and the rectal stump with a solution of mercury perchlorate, getting, in a uncontrolled study, a decrease in the rate of AR from 14% to 2% [28]. This dramatic reduction of the percentage of relapses after irrigation was confirmed at St. Mark’s Hospital, where, among 400 patients, the rate was only 1–2% [28].

The intestinal preparation with PEG solution seems to reduce the risk of the presence of exfoliated cells in the colonic lumen. In a recent study [29], intestinal preparation before right hemicolectomy allowed breaking down of the amount of luminal viable neoplastic cells.

Between 2000 and 2015, 147 right hemicolectomies were carried out in our unit. Mechanical surgical staplers were used in 25 cases (17%); most of the anastomoses were hand-sewn using slowly absorbable sutures. Intestinal stump draping was performed in only 10% of all treated cases. We registered only two cases of AR (1.3%). Both of them belonged to the manual anastomosis group. In the first case, 13 months after surgery, a flat, 1-cm lesion was found; in this case, polyvinylpyrrolidone had not been used. The second case is our patient reported in the present article.

Undoubtedly, an anastomosis could be considered an area of great proliferative instability that can influence the development of a neoplastic relapse. Several experiments carried out in animals have proved the role of these sites of proliferative stimulation in carcinogenesis, when a carcinogen is administered [11, 30].

Whenever a neoplastic recurrence on the anastomotic line occurs in the presence of negative intestinal margins, as usual in right colectomies, the implantation of neoplastic cells or a change in the biology of the tumor could be the possible causes. In our patient’s case, the hypothesis of a neoplastic polypoid lesion missed during preoperative workup should be ruled out, because both intestinal stumps were completely exempt from neoplastic infiltration, and the presence of an adenomatous lesion was excluded by three colonoscopies performed by experienced professionals. Endoscopic follow-up can detect chronic signs of phlogosis, with occasional presence of ulcers, even more than 24 months after creation of the anastomosis. Possible areas of hyperemia, edema, and sometimes microerosions into the surgical site a few months after operation shall be considered a normal condition that will disappear within 6 months. According to data, in about 90% of cases, the recurrence can be found through endoscopy; this percentage can reach 100% by means of endoscopic ultrasound [31].

A metachronous cancer can be excluded due to the relative earliness of the lesion detected a few months after surgery. At 2 months, a small area of hyperemia and edema with microerosions was found. Such a situation, especially when it involves all or most of the anastomotic circumference, could be considered as normal; in our patient’s case, it was only “strange” – in its small extension and hard consistency.

Lymph node metastases can also be excluded due to the good number of lymph nodes isolated. Furthermore, histology carried out on each of them demonstrated the absence of tumoral cells.

Surgery was carried out following scientifically proved guidelines and standard techniques: Section lines were far enough from tumoral mass; intestinal stumps were draped with gauzes soaked with a water-based solution of polyvinylpyrrolidone 5% for 15 minutes; and 4 L of PEG solution were administered orally the night before surgery. All measures that were appropriate to avoid the implantation of exfoliated cells were adopted.

Refusal of adjuvant chemotherapy could be considered a crucial event for the development of AR: Taking into account that treatment usually starts 30–45 days after surgery and that the AR was diagnosed 60 days after surgery, we can affirm that missed chemotherapy could not have avoided carcinogenesis.

To the best of our knowledge, the implantation of exfoliated neoplastic cells on anastomotic lines seems to represent the sole mechanism able to explain neoplastic AR. As already remarked by other authors [32, 33], tumoral cell dissemination during surgery could represent the most important factor involved in AR. Up to now, advancements in technique and technologies have brought increasingly significant improvements, allowing recognition of increasingly small and hidden mucosal lesions, but little has been done to avoid neoplastic cell dissemination. Of course, technical precautions could limit this unpleasant phenomenon, but much effort is still required to clear cases.

## Data Availability

All data generated or analyzed during this study are included in this published article.

## Abbreviations

AR: Anastomotic recurrence
PEG: Polyethylene glycol
SCCR: Synchronous colorectal cancer
